# Commencement of New Volume

**Published:** 1867-08

**Authors:** 


### Editorial Department.

### Commencement of New Volume.

It will be noticed that the present number is the first of a new volume, and naturally offers an opportunity to invite the coöperation and support of the profession. It is not wholly from selfish interest in the Journal that we would urge upon physicians an effort to promote the science and enlarge the knowledge of true medicine. Every careful observer of the status of the profession in this country, has noticed how little thought the masses of the profession actually bestow upon either the principles or philosophy of cure in medicine—how thoroughly contented they are to even imperfectly understand the symptoms of common diseases, and the usual routine of treatment applicable to them, without ever attempting to add to the general fund of knowledge, or even becoming acquainted with the recent and important discoveries which others have added to what was known of disease and its modes of treatment. After obtaining honorable admittance to the ranks of the profession, all further effort is directed to the one absorbing object of business, pursuing it with commendable zeal, but almost entirely forgetting the higher and truer objects of professional ambition. True success with a physician cannot be supposed to consist in obtaining a large professional income, and it is taking a very incorrect and unworthy view, to regard success in getting business, as any truthful standard of merit. To expose error, or to discover truth, are just objects of professional pride; to perfectly understand what really is known of practical value to physicians is truly commendable, or to add to the amount of well observed and carefully recorded facts, usually shows a disposition to promote the general good, and an active interest in the progress of our art. We believe that no country can furnish a larger proportion of active, intelligent and earnest men, than are now engaged in the practice of medicine in the United States, and that our own vicinity will bear creditable comparison in this respect. Buffalo sustains an enviable reputation, and is not surpassed by any city on the globe, considering its size, for the amount or quality of contributions both periodical and standard to the medical literature of the world. If our reputation in this respect, due largely to men who now occupy other places, can be sustained in the future, we shall have abundant ground for honest pride.

Buffalo Medical and Surgical Journal commences its seventh volume looking confidently to its friends for the choicest material to fill its pages, believing that it will increase in usefulness, and grow in favor, mainly through the partiality of the profession whose interests it desires to promote. It is not the organ of any locality or individual, but is open to the whole profession for the fullest expression of thought, and is earnestly commended to the favorable notice of all who have well considered opinions to express, or would promote a knowledge of the truth in medicine. Sometimes it is intimated that a medical journal belongs to the editor and proprietor and is dependent upon him for success. While be believe that upon his *fair-*

ness, fidelity and faithfulness to the interests of others, may largely depend the success of such enterprise, still we would also have physicians understand that it belongs to the whole of the medical profession, and its success or failure indicates rather the *status* of the profession than the ability of those who conduct its passage through the press. If the medical journal of a city is devoid of interest and of doubtful value, the profession are generally devoid of intelligence and their modes of practice unscientific and obsolete; it is in as great degree representative of the medical public.

It is with feelings of great satisfaction that we enter upon our new volume, and commence its labors. The interest manifested in its pages, and the support it is now so generously receiving from the profession are sources of pride and pleasure, and we have to thank our contributors and patrons in behalf both of ourselves and the profession for their efforts and aid, and to cordially and earnestly invite their continued support. Relying confidently upon this, we anticipate increasing prosperity and strength, and pledge our best efforts to deserve it.

